# Degradation of connexin 50 protein causes waterclefts in human lens

**DOI:** 10.1515/med-2020-0249

**Published:** 2020-11-17

**Authors:** Yosuke Nakazawa, Teppei Shibata, Noriaki Nagai, Eri Kubo, Hiroomi Tamura, Hiroshi Sasaki

**Affiliations:** Division of Hygienic Chemistry, Faculty of Pharmacy, Keio University, 1-5-30 Shibakoen, Minato-ku, Tokyo 105-8512, Japan; Department of Ophthalmology, Kanazawa Medical University, 1-1 Daigaku Uchinada-machi, Kahoku-gun, Ishikawa 920-0293, Japan; Laboratory of Pharmaceutical Technology, Faculty of Pharmacy, Kindai University, 3-4-1, Kowakae, Higashiosaka City, Osaka 577-8502, Japan

**Keywords:** cataract, waterclefts, adhesion molecules

## Abstract

Cataracts are mainly classified into three types: cortical cataracts, nuclear cataracts, and posterior subcapsular cataracts. In addition, retrodots and waterclefts are cataract subtypes that cause decreased visual function. To maintain an orderly and tightly packed arrangement to minimize light scattering, adhesion molecules such as connexins and aquaporin 0 (AQP0) are highly expressed in the lens. We hypothesized that some main and/or subcataract type(s) are correlated with adhesion molecule degradation. Lens samples were collected from cataract patients during cataract surgery, and mRNA and protein expression levels were measured by real-time RT-PCR and western blotting, respectively. The mRNA levels of adhesion molecules were not significantly different among any cataract types. Moreover, AQP0 and connexin 46 protein expressions were unchanged among patients. However, connexin 50 protein level was significantly decreased in the lens of patients with WC cataract subtype. P62 and LC3B proteins were detected in the WC patients’ lenses, but not in other patients’ lenses. These results suggest that more research is needed on the subtypes of cataracts besides the three major types of cataract for tailor-made cataract therapy.

## 1 Introduction

Cataracts are the leading cause of blindness in the world despite the success of surgical replacement with artificial lenses [1,2]. Adhesion molecules are highly expressed in the lens to prevent light scattering and keep the lens transparent. They make the adhesions tight among the lens cells.

Gap junctions play an important role in cell adhesion and intracellular communication among cells. They are oligomeric assemblies of members of a family of related proteins called connexins. Connexins belong to a family of proteins with four transmembrane domains, including at least 24 different members in humans [3]. At least three of these connexins are expressed in the lens: connexin 43 (Cx43; known as GJA1, α1 connexin), connexin 46 (Cx46; known as GJA3, α3 connexin), and connexin 50 (Cx50; known as GJA8, α8 connexin). The lens is composed of two types of cells: epithelial and fiber cells. The epithelial cells form a monolayer that covers the anterior surface of the lens, extending from the anterior pole to its equatorial surface. The fiber cells compose the bulk of the lens and are elongated. Epithelial cells express Cx43, whereas fiber cells express Cx46 and Cx50. In the lens fiber cell membrane, Cx50 and Cx46 proteins account for 12.9% and 2.1% of the proportion, respectively [4]. Mutations in connexins are known to cause cataracts [5,6], and fiber cells with cortical opacities have degenerated gap junctions [7].

Aquaporin 0 (AQP0) is a member of the aquaporin family, whose members act as water permeability channels. AQP0 has cell-to-cell adhesion [8,9]. It is the most abundant protein in lens fiber cells, accounting for more than 30% of the total membrane protein. The decrease in cell adhesion function of AQP0 is known to cause cataract [10,11]. In the fiber cell integral membrane, there are more than 75% adhesion-related proteins [4].

**Figure 1:**
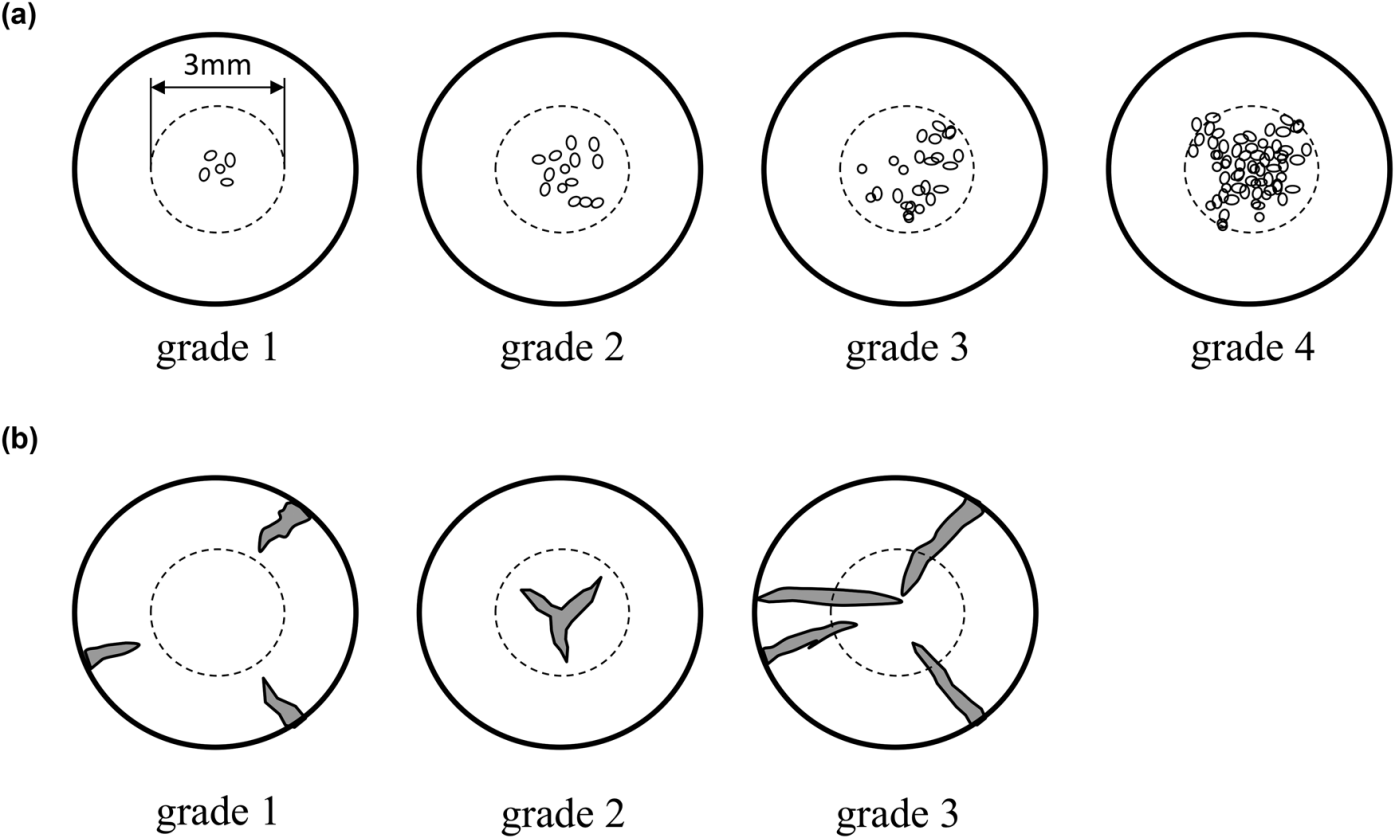
Images of RDs and watercleft cataract classification. (a) RD classification. RD grade 1 was defined as fewer than 4 small dots observed within a central 3 mm zone of the pupil. RD grade 2 was defined as more than 5 dots or large dots but observed less than 25%, and RD grade 3 was defined as dots observed 25–50% within a central 3 mm zone of the pupil. RD grade 4 was defined as many dots (more than 50%) within a 3 mm zone of the pupil. (b) WC classification depending on the location of WC in relation to the central 3 mm zone of the pupil. WC grade 1 was defined as those observed outside of the 3 mm zone of the pupil. WC grade 2 was defined as those observed within a 3 mm zone. WC grade 3 was defined as those observed both in a 3 mm zone of the pupil and outside.

Cataracts are mainly classified into three types based on the section of the lens that has become opaque: cortical cataract (COR), nuclear cataract (NUC), and posterior subcapsular cataract (PSC). COR has opacity in the outer section of the lens, NUC in the inner core section, and PSC in the superficial region below the capsule on the posterior side. In addition to the three major types of cataract, retrodots (RD) and waterclefts (WC) are strongly associated with the impaired visual activity [12,13]. RD and WC are well correlated with the three major types of cataracts. Miyashita et al. designed a classification of RD and WC according to the situation and the number or the size of dots (Kanazawa Medical University Cataract Classification and Grading System; KMUCCGS: Figure 1) [14].

In this study, we tried to elucidate a causal relationship between cataract types and cell-to-cell adhesion molecule degradation in lens, and the contribution of cell adhesion molecules to the cataract development. AQP0, Cx46, and Cx50 expression levels were investigated in COR, NUC, and PSC in addition to RD and WC.

## 2 Methods

### 2.1 Ocular examination

Thirteen eyes of 13 cataract patients (72.8 ± 8.6 years, 6 males and 7 females) who underwent cataract surgery in Kanazawa Medical University Hospital from June 2015 to August 2015 were enrolled in this study. Cataracts were diagnosed by one ophthalmologist from slit-lamp images of the right eyes obtained under maximal mydriasis and classified into the three major types (COR, NUC, and PSC) using the WHO classification system [15] and into the two subtypes (RD and WC) using KMUCCGS [14] (Table 1).

**Table 1:** Patient characteristics. COR, NUC, and PSC were graded using the WHO simplified cataract grading system, and RD and WC were graded using KMUCCGS [14]

Patient ID	Age (years) and sex	Cataract types and grade
#1	71, female	COR3
#2	71, male	NUC2
#3	60, male	PSC3
#4	71, male	RD3
#5	72, female	WC3
#6	54, female	NUC1
#7	80, male	NUC1 + RD3
#8	78, male	NUC1 + WC3
#9	70, male	COR3
#10	71, female	NUC2
#11	84, female	COR3 + NUC2
#12	80, female	COR2 + NUC2 + RD2
#13	84, female	COR2 + NUC2 + WC3

This study was approved by the institutional review board of Kanazawa Medical University (Approval code: G101) with the appropriate informed consent obtained from all patients. Methods for securing human tissues comply with the tenets of the Declaration of Helsinki (2013).

### 2.2 Cataract surgery

All the cataract surgeries were done by one experienced surgeon (HS) using the standard ultrasound phacoemulsification cataract surgery. Lens fiber cells of cataract patients were collected using the cassette pack of CENTURION VISION SYSTEM (Alcon Japan Ltd, Tokyo, Japan). Fiber cells solution were stored at −80°C until further use.

### 2.3 Real-time PCR

Lens fiber cell samples were transferred from surgery bags to centrifuge tubes and collected by centrifugation at 1,000 × *g* for 20 min at 4°C. Total RNA was isolated using TRIzol Reagent (Invitrogen, Gaithersburg, MD, USA) and reverse transcribed using SuperScript II Reverse Transcriptase kit (Invitrogen, Carlsbad, CA, USA) according to the manufacturer’s recommendations. The real-time PCR analysis was performed using SYBR-Green PCR Master Mix (Applied Biosystems, Foster City, CA, USA). The expression levels of individual genes were normalized to β-actin levels and are shown relative to the control samples as indicated. PCR primer sequences are listed in Table 2.

**Table 2:** Primers used for real-time PCR analysis

Primer (accession ID)		Sequences (5′–3′)
*AQP0*	FOR	GGAGGGCCATATTCGCTGAG
(NM_012064)	REV	CCAAGCCAAATGCCATAGCC
*Cx50*	FOR	GAAGATCAGCACAGGACCCC
(NM_005267.5)	REV	GATCGTCTGACCTGGCTCG
*Cx46*	FOR	TGGAAGAAGCTCAAGCAGGG
(NM_021954.4)	REV	TTGTAGAGCTTGGCGGACTG
*Beta-actin*	FOR	AGAAGGATTCCTATGTGGGCG
(NM_001101.5)	REV	GGATAGCACAGCCTGGATAGCA

### 2.4 Western blot analysis

Lens fiber cell samples were transferred from surgery bags to centrifuge tubes and centrifuged at 1,000 × *g* for 20 min at 4°C. The pellets were homogenized in ice-cold phosphate-buffered saline with a protease inhibitor cocktail (Sigma-Aldrich), and the homogenates were centrifuged at 20,000 × *g* for 20 min. The fiber cell membrane pellets were resuspended in the SDS sample buffer, and samples were separated on 10% SDS gels and transferred to nitrocellulose membranes. Western blots were performed using an anti-AQP0 antibody [16], an anti-Cx46 antibody (C-20; Santa Cruz, Santa Cruz, CA), an anti-Cx50 antibody (B-11; Santa Cruz), an anti-P62 antibody (GP62-C; PROGEN), LC-3B (#2,775; Cell signaling technology), and an anti-β-actin antibody (C-11; Santa Cruz). The intensity of each band was quantified by Image-J software, and the band intensity of each proteins was normalized with band intensities of β-actin.

### 2.5 Statistical analysis

All data are reported as the mean ± standard error. Statistical analysis of the data was done using the Aspin–Welch’s *T*-test using SPSS statistic software (version 24 for Macintosh, IBM Inc.). *P* values less than 0.05 indicated statistical significance.

## 3 Results

### 3.1 mRNA expressions in cataract patients

We measured the mRNA expression of cell adhesion-related genes AQP0, Cx50, and Cx46, in each patients’ lens. Beta-actin was also measured as an internal control. The patient characteristics are listed in Table 1.

**Figure 2:**
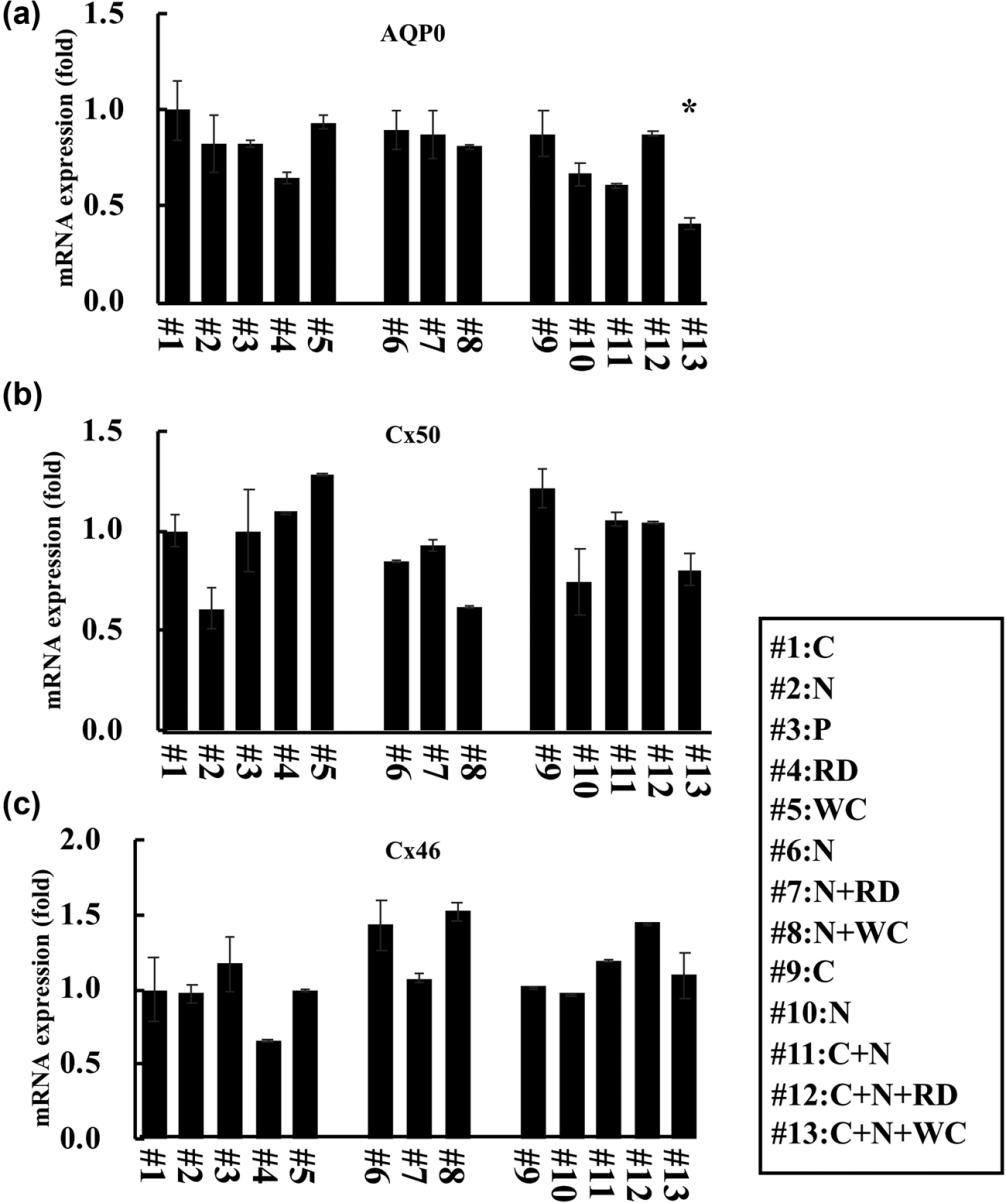
mRNA expression in several types of cataract patients. (a) AQP0 mRNA expression, (b) Cx50 mRNA expression, and (c) Cx46 mRNA expression in several types of cataract lens was measured by real-time PCR. C: COR patient, N: NUC patient, P: PSC patient, RD: retrodots cataract patient, WC: watercleft cataract patient, N + RD: NUC with RDs, N + WC: NUC with WC, C + N: COR with NUC, C + N + RD: COR with nuclear and RDs cataracts, and C + N + WC: COR with nuclear and watercleft cataracts. Beta-actin was used as an internal control. This experiment used 3–4 independent samples per groups, and each bar indicates the mean ± SD. Statistical analysis of the data was done using the Aspin–Welch’s *T*-test, and the asterisk (*) indicates a *p* < 0.05 compared to COR lens (#1 patient).

In AQP0 mRNA expression, there were no significant differences in all cataract patients except patient #13, who has a mixed type of cataract with COR, NUC, and WC (Figure 2a). There were no significant differences in Cx50 mRNA and Cx46 mRNA expression in all cataract patients (Figure 2b and c).

### 3.2 Protein expressions in cataract patients

**Figure 3:**
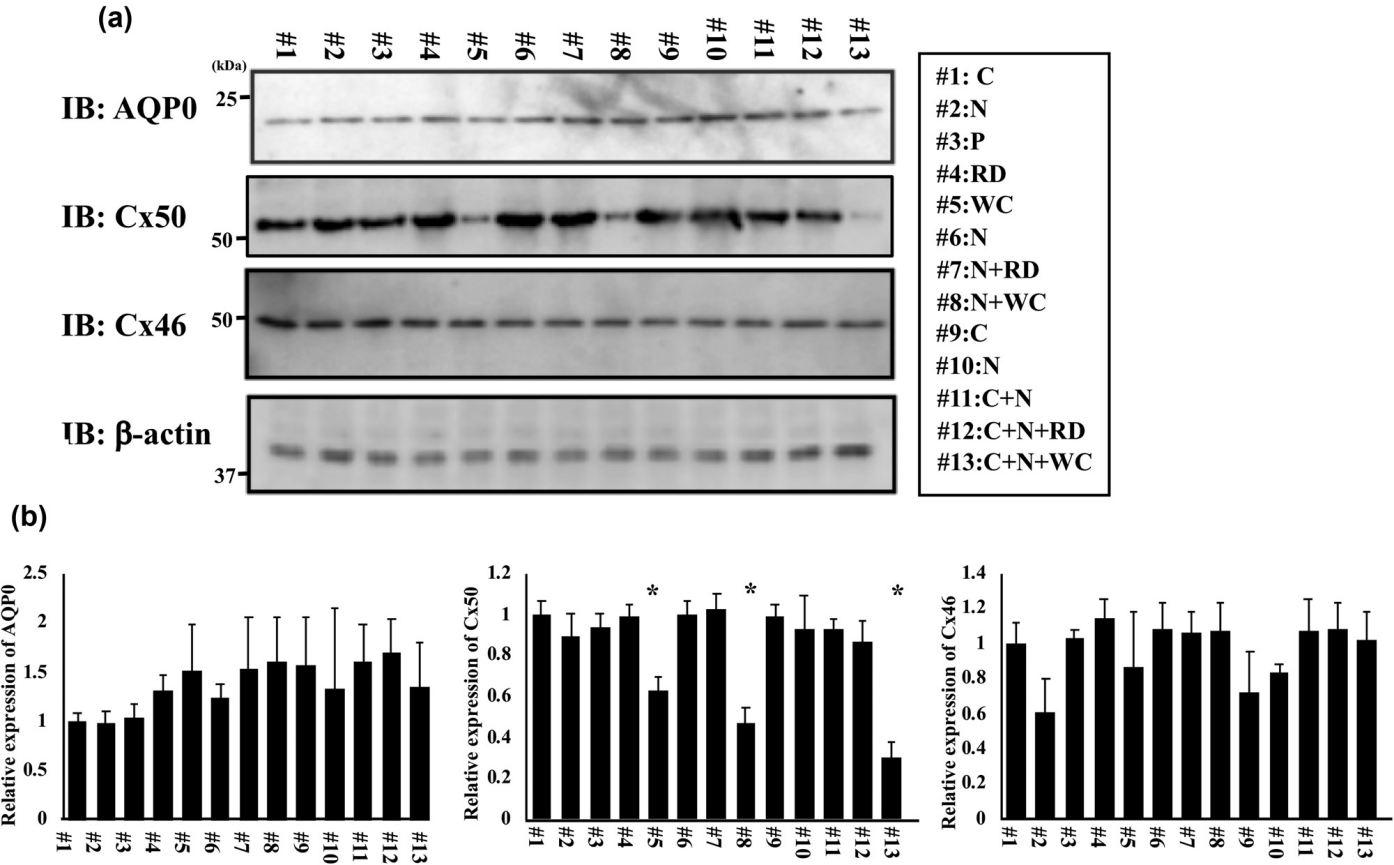
Protein level in cataract patients. (a) AQP0 protein level, Cx50 protein level, Cx46 protein level, and β-actin protein as an internal control were analyzed by western blot using anti-AQP0 antibody, anti-Cx50 antibody, and anti-Cx46 antibody, and β-actin antibody, respectively. C: COR patient, N: NUC patient, P: PSC patient, RD: retrodots cataract patient, WC: waterclefts cataract patient, N + RD: NUC with retrodots, N + WC: NUC with WC, C + N: COR with NUC, C + N + RD: COR with nuclear and RDs cataracts, and C + N + WC: COR with nuclear and WC cataracts. (b) Band intensities were quantified using NIH-ImageJ software. This experiment was used 3–4 independent samples per groups, and each bar indicates the mean ± SD. Statistical analysis of the data was done using the Aspin–Welch’s *T*-test, and the asterisk (*) indicates a *p* < 0.05 compared to COR lens (#1 patient).

Next, we measured the AQP0, Cx50, and Cx46 protein levels in several cataract patients. The AQP0 expression in lens of patient #13 (COR + NUC + WC) was lower compared with other cataract patients (Figure 3a, upper panel).

Cx50 protein was decreased in the cataract lens that had WC (patients #5, #8 and #13) (Figure 3a, middle panel). There were no differences in Cx46 protein among cataract patients (Figure 3a, lower panel). The intensity of each band was quantified by Image-J software, and the band intensity of each protein was normalized with band intensities of β-actin (Figure 3b). These results suggested that a decrease in Cx50 protein caused lens WC.

### 3.3 LC3B and p62 expressions in cataract patients

**Figure 4:**
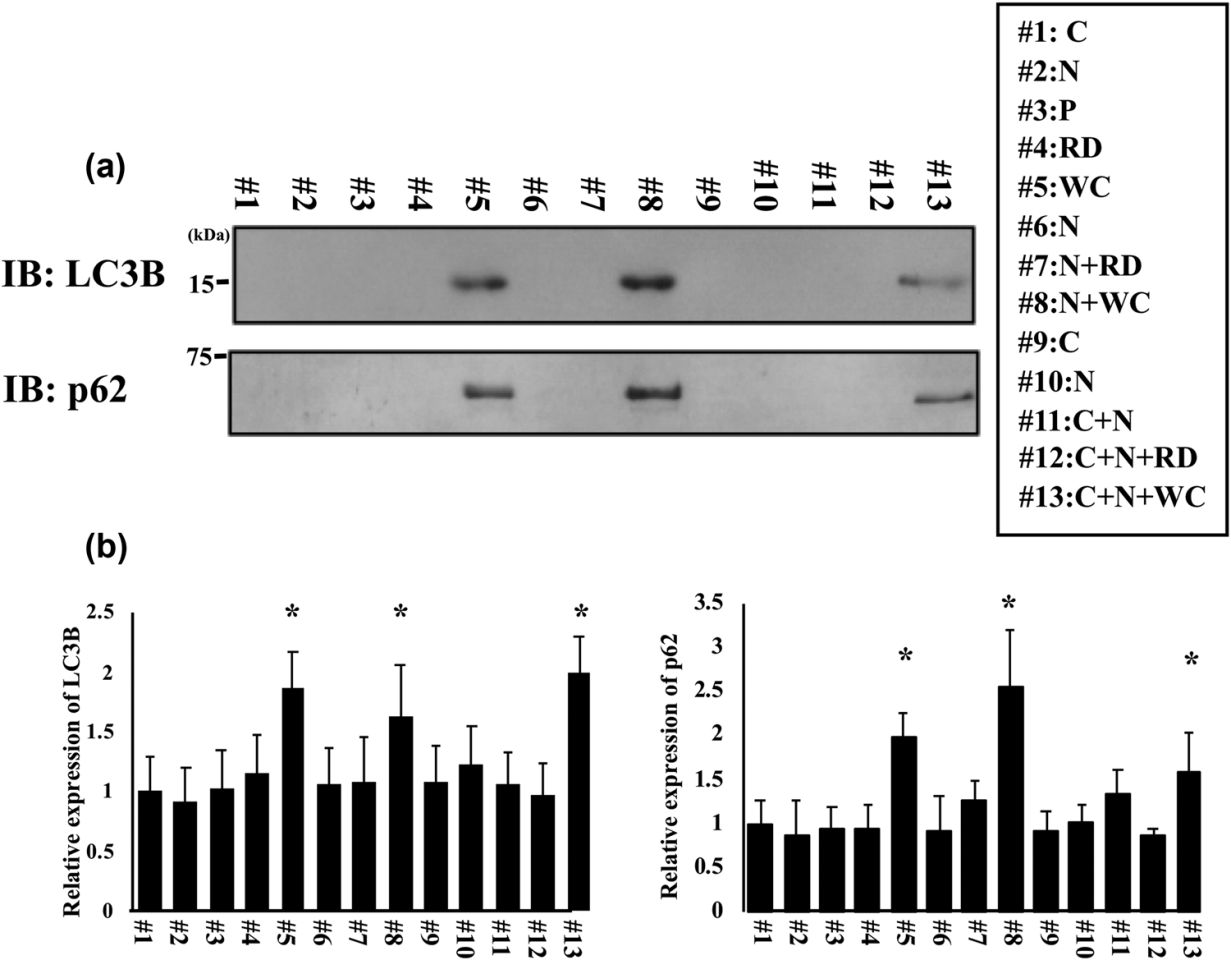
Protein levels were analyzed in cataract patients. (a) LC-3 protein level and p62 protein level were analyzed by the western blot analysis. C: COR patient, N: NUC patient, P: PSC patient, RD: retrodots cataract patient, WC: waterclefts cataract patient, N + RD: NUC with RDs, N + WC: NUC with WC, C + N: COR with NUC, C + N + RD: COR with nuclear and RDs cataracts, and C + N + WC: COR with nuclear and WC cataracts. (b) Band intensities were quantified using NIH-ImageJ software. This experiment was used 3–4 independent samples per groups, and each bar indicates the mean ± SD. Statistical analysis of the data was done using the Aspin–Welch’s *T*-test, and the asterisk (*) indicates a *p* < 0.05 compared to COR lens (#1 patient).

Cx50 was reported to be co-expressed with LC3 and degraded by autophagy in HeLa cells [17]. During autophagy, the cytosolic form of LC3 (LC3-I) is conjugated with substrates to form a LC3-phosphatidylethanolamine conjugate (LC3-II), which is recruited to autophagosomal membranes. The protein p62, also known as SQSTM1, was known to interact with autophagic substrates and delivers them to autophagosomes for degradation. We measured the LC3B and p62 expression in cataract patients. Based on the results, LC3B protein could be detected only in the samples with WC (patients 5, 8, and 13) (Figure 4a). The band intensity was measured by Image-J software (Figure 3b). These results suggest that Cx50 protein could be degraded by autophagy in cataract lens, and downregulation of gap junction function weakens the adhesion among cells, which developed the WC in lens.

## 4 Discussion

It has been estimated that if the onset of cataract is delayed by 10 years, and the number of cataract patients would decrease by 45% [18]. Therefore, investigating the pathogenetic mechanism of cataract formation is important for delaying or preventing cataract formation without surgical methods. In this study, we showed, for the first time, that the protein levels of Cx50 were lower in lenses with WC and Cx50 and could be degraded by autophagy.

The Melton eye study was an English community-based epidemiological study that revealed that RD and WC were present in 11% and 17% of participants, respectively. They were classified using the Oxford Clinical Cataract Classification and Grading System [19]. In this study, we classified the RD and WC grades by KMUCCGS depending on the number or the size of dots in the lens. In the Han Chinese general population, among three climatically different places (Sanya, Hainan, and Taiyuan, Shanxi, of China and Taichung, Taiwan), and in patients older than 40 years, the prevalence of RD was 16.2–40.7%, while it was 4.7–11.9% for WC [14]. According to the aforementioned studies, the two subtypes of cataracts are prevalent in the general population. We have reported that WC was associated with the decreased lens power, causing hyperopia, decreased visual acuity, and higher straylight, and that such lenticular change should be considered for surgery [13]. Although WC is very common in the elderly population and can cause severe deterioration of visual function, the significance of the impact on visual function is not widely understood. The risk factors of WC are still unclear. Durant et al. reported that the total number of analgesics used in the past year and total lifetime sunlight exposure were significantly associated with WC [20]. Conversely, Miyashita et al. reported that lifetime ultraviolet exposure to the eye is negatively correlated with WC [14].

In this study, we examined the protein level and mRNA expression of AQP0, Cx46, and Cx50 in cataract lens to clarify the correlation between cataracts and cell adhesion molecules. Cx50 protein levels were decreased in the patients with WC. However, Cx50 mRNA expression did not changed compared to other cataract patients. We hypothesize that degradation of Cx50 protein in lenses with WC was progressive, such as autophagy and/or proteasome-ubiquitin systems. It was reported that Cx50 could co-express with LC3 in HeLa cells and are degraded by autophagy [17]. We checked LC3 protein expression using the western blot analysis. LC3 and p62 protein were detected only in WC patients with or without other cataract types. Upon induction of autophagy, a portion of the cytoplasm was enclosed by a phagophore (isolation membrane), which became the double-membrane structure of the autophagosome. The outer membrane of the autophagosome connects with late endosomes and lysosomes to form the autolysosome. The autolysosome-enclosed materials are degraded by lysosomal hydrolases. The degradation products are recycled to the cytosol [21]. In our present study, LC3 and p62 proteins were detected only in WC patients with or without other cataract types (Figure 4). In the αB-crystallin (R120G) mutation mouse model, cataracts lead to an increase in αB-crystallin aggregation, which induces lens opacity with aging [22]. In this murine lens, the number of autophagosomes without any features of degradation increases, and p62-positive aggregates also accumulate. These data suggested that autophagy was blocked at a late step in the mutant lens. The autophagy activity could measure not only just the increase in the autophagy protein levels but also the autophagy flux using lysosome inhibitor [23]. It will need to further investigate the autophagy flux and the relationship between autophagy in the lens and the cataract development.

Connexins compose the gap junction. These gap junctions are highly specialized clusters of intercellular channels that form where the membranes of two neighboring cells are closely apposed [24]. In the lens, Cx43 and Cx50 are expressed in epithelial cells, and it was reported that Cx43 and Cx50 could not form homomeric/heterotypic junctions (formed by the docking of hemichannels composed of different connexins). However, they can form the heteromeric/heterotypic junction (formed by the mixing of two different connexins within a hemichannel) as studied in Xenopus oocytes expressing Cx43 and Cx50 pairs [25,26]. Since Cx43 protein is not expressed in the fiber cells, the epithelial–fiber cell connection could be through either homotypic Cx50 channels or heterotypic/heteromeric channels of Cx43 and Cx50. Therefore, the decrease in the Cx50 protein level may have caused weakening of the connection of epithelial–fiber cells.

In the fiber cells, Cx46 and Cx50 are expressed and compose the gap junction between them. Cx46 and Cx50 could form both the heterotypic and heteromeric/heterotypic junction [27,28]. Since there is low Cx46 expression in cortical fiber cells and high expression in the nuclei of lens, cell adhesion in cortical fiber cells depends heavily on Cx50 protein. A decrease in Cx50 protein leads to the failure of regular cell-to-cell appositions, leading to vacuoles in epithelial–fiber cells and/or fiber–fiber cell cortical region connections, and causes WC. Thus, WC does not supervene upon NUCs but on CORs. It was reported that gap junctions of Cx50 knockout mice lens were four times shorter than those of wild-type or Cx46 knockout mice [29]. It was also reported that transgenic overexpression of Cx50 induced smaller lenses and central cataract [30]. Conversely, Cx50 knockout mouse showed the smaller lens and intercellular spaces of various sizes in lenses [31]. From these reports, Cx50 expression levels in lens are important for lens cell-to-cell adhesion and transparency. These results suggest that the Cx50 protein expression changes caused the smaller gap junctions, downregulation of gap junction functions, and weakened the adhesion among the cells, causing the development of WC in lens. We have checked DNA sequences, but we could not find any differences. To the best of our knowledge, there are no reports about polymorphisms and/or secondary modification of Cx50 in WC samples. These modification studies need further investigation.

In lens fiber cells, AQP0 are important proteins for cell-to-cell adhesion, aside from connexins. In the AQP family, AQP1 and AQP5 are expressed in the epithelial cells, while AQP0 and AQP5 are expressed in the fiber cells. AQP0 is known to have cell-to-cell adhesion properties. However, AQP1 and AQP5 do not have this function [8,9,32]. AQP0 and M23-AQP4 (i.e., the splicing variant of AQP40) are reported to have cell-to-cell adhesion properties [33]. We tried to detect the changes in AQP4 expression in this study, but we were unsuccessful (data not shown).

In patient 13 (COR + NUC + WC), AQP0 mRNA and protein expression were decreased. We analyzed nucleotide sequences in the promoter region of AQP0 (−947 to +1). There were no mutations in this region of AQP0 (data not shown). It was reported that AQP0 expression is downregulated in transgenic mice overexpressing insulin-like growth factor (IGF-1) and that IGF-1 is downregulated in diabetic patients [34,35]. It was also reported that AQP0 expression is increased in diabetic rats induced by streptozotocin [36]. These data suggested that patient 13 might be hypoglycemic and/or have increased lens IGF-1 protein expression and that IGF-1 regulated the AQP0 mRNA expression. Unfortunately, we could not get the patients’ medical history due to our use of anonymous sampling.

The C-terminal end AQP0 could interact directly with two binding sites within the intracellular loop of Cx50, but cannot with Cx46 [37]. It was also reported that Cx50 did not affect AQP0 expression in a double knockout study of Cx50 and Cx46 [5]. Therefore, it was suggested that the decreases in AQP0 mRNA and protein expression were independent of those in Cx50 protein expression.

The main limitations of this study are failure to include the patients’ conditions, the study’s small sample size, and the use of cell mixture samples. We were not able to follow the medical history of the patients, such as their blood biochemistries and basal diseases. Thus, we were not able to check other phenotypes in this study, such as patients’ lens conditions due to other diseases such as diabetes. In addition, we used the lens cells collected in the cassette pack after cataract surgery. Thus, in many samples, protein and/or mRNA concentrations were too low, and the cassette packed samples included both epithelial cells and fiber cells of the lens. It may be valuable to condense the protein or mRNA and separate the epithelial cells and fiber cells. Although samples used in this study were a mixture of epithelial cells and fiber cells, the bulk of the lens cells were fiber cells, and Cx50 and AQP0 were fiber cell-specific proteins. Thus, we consider it to have had little effect on the study’s results.

In conclusion, our study found that protein levels of Cx50 were decreased in lenses with WC. We also found that Cx50 could be degraded by autophagy. Additional studies for not only the major three types of cataracts but also for the subtypes of cataracts (RD and WC) are needed to improve tailor-made cataract treatment and prevent cataract formation to decrease the use of surgery.
